# Real-World PSA Response and Safety Outcomes of 177Lu-PSMA-617 in Heavily Pretreated Metastatic Castration-Resistant Prostate Cancer: A Single-Center Retrospective Validation Study

**DOI:** 10.3390/jcm15207766

**Published:** 2026-10-09

**Authors:** Jin Hyeok Choi, Chang Gon Kim, Kyung Tak Oh, Ji Eun Heo, Jongsoo Lee, Won Sik Jang, Seung Hwan Lee, Young Deuk Choi, Seung Hoon Beom, Won Jun Kang, Woong Kyu Han, Hyunho Han

**Affiliations:** 1 Department of Urology, Urological Science Institute, College of Medicine, Yonsei University, Seoul 03722, Republic of Korea; chj89@yuhs.ac (J.H.C.); okt1226@yuhs.ac (K.T.O.); heoji87@yuhs.ac (J.E.H.); js1129@yuhs.ac (J.L.); sindakjang@yuhs.ac (W.S.J.); leeseh@yuhs.ac (S.H.L.); youngd74@yuhs.ac (Y.D.C.); 2 Division of Medical Oncology, Department of Internal Medicine, Yonsei Cancer Center, College of Medicine, Yonsei University, Seoul 03722, Republic of Korea; inspector@yuhs.ac (C.G.K.); beomsh@yuhs.ac (S.H.B.); 3 Department of Nuclear Medicine, Severance Hospital, College of Medicine, Yonsei University, Seoul 03722, Republic of Korea; mdkwj@yuhs.ac

**Keywords:** 177Lu-PSMA-617, metastatic castration-resistant prostate cancer, prostate-specific membrane antigen, radioligand therapy, progression-free survival, real-world evidence, safety

## Abstract

**Background/Objectives:** 177Lu-PSMA-617 is an established standard-of-care treatment for eligible patients with prostate-specific membrane antigen (PSMA)-positive metastatic castration-resistant prostate cancer (mCRPC). Treatment schedules and follow-up in routine practice may differ from clinical trial protocols. Post-approval evaluation is needed to determine whether trial-reported prostate-specific antigen (PSA) responses and adverse events are also observed outside trials. We assessed outcomes in heavily pretreated patients receiving 177Lu-PSMA-617. **Methods:** This retrospective study included 13 patients treated outside clinical trials at Severance Hospital, with follow-up through 30 June 2026. PSA50 and PSA90 denoted unconfirmed reductions of at least 50% and 90% from pretreatment baseline. Study-defined progression-free survival (PFS) included radiographic progression, a PSA increase of at least 50% from baseline considered disease progression, or death. **Results:** Median age was 69 years. All patients had received androgen-deprivation therapy, an androgen receptor pathway inhibitor, and docetaxel. Median baseline PSA was 88.79 ng/mL, and the median number of cycles was four. PSA50 and PSA90 occurred in six (46.2%) and three (23.1%) patients, respectively. Eight patients had a PFS event; median study-defined PFS was 5.9 months (95% confidence interval (CI), 1.4 months to not estimable). Two patients required activity reduction for cytopenias. Dry mouth was recorded in five patients (38.5%). **Conclusions:** PSA responses in this cohort support the applicability of trial findings to routine care. Documented adverse events included recognized toxicities. The PSA-inclusive PFS endpoint differs from trial radiographic PFS. Small sample size, concomitant treatment, and retrospective ascertainment limit interpretation. Larger cohorts with standardized follow-up are needed to assess response durability.

## 1. Introduction

Metastatic castration-resistant prostate cancer (mCRPC) progresses despite castrate serum testosterone levels. Treatment after an androgen receptor pathway inhibitor (ARPI) and taxane chemotherapy depends on previous exposure, disease distribution, performance status, organ function, and actionable molecular alterations. Although several therapies prolong survival, cumulative treatment toxicity and advancing disease restrict subsequent options [1]. Treatment outcomes must also be interpreted in relation to the manifestation of progression being measured; changes in prostate-specific antigen (PSA), imaging findings, and symptoms are not interchangeable [2].

Prostate-specific membrane antigen (PSMA) provides a target for delivering lutetium-177 radiation to PSMA-expressing lesions. The phase III VISION trial established the benefit of 177Lu-PSMA-617 in PSMA-positive mCRPC after ARPI and taxane exposure. Compared with protocol-permitted standard care alone, 177Lu-PSMA-617 plus standard care improved imaging-based progression-free survival (PFS; median, 8.7 vs. 3.4 months) and overall survival (15.3 vs. 11.3 months) [3]. These findings supported regulatory approval in this treatment setting [4].

Other randomized trials have defined its activity in different clinical settings. In TheraP, 177Lu-PSMA-617 produced a higher rate of PSA reductions of at least 50% (PSA50) than cabazitaxel in docetaxel-pretreated patients selected using PSMA and fluorodeoxyglucose (FDG) positron emission tomography (PET) [5]. PSMAfore subsequently demonstrated longer radiographic progression-free survival than a change in ARPI in taxane-naive mCRPC [6]. Its final intention-to-treat analysis did not show a statistically significant overall-survival difference, in the setting of substantial crossover [7]. These trials provide the clinical context for evaluating outcomes after routine adoption of 177Lu-PSMA-617.

Evidence outside randomized trials is also substantial. Early multicenter studies and a prospective phase II study documented biochemical activity in heavily pretreated patients [8,9]. Subsequent reports described outcomes over repeated cycles and longer follow-up [10,11,12], and a systematic review summarized the growing clinical experience [13]. The prospective REALITY registry provided longitudinal data from 254 patients [14]. More recent multicenter and post-approval series have reported treatment responses under routine-care conditions [15,16,17,18].

Post-approval evaluation remains necessary because treatment delivery and follow-up in routine care may differ from clinical trial protocols. Patients may receive fewer cycles, treatment intervals may vary, and PSA measurements and imaging may not occur at fixed times. Continued assessment is needed to determine whether the PSA responses and adverse events reported in trials are also observed under these conditions. In one post-approval series, both favorable response and disease progression led to treatment delays or discontinuation [17].

We therefore conducted a single-center retrospective validation study of 13 heavily pretreated patients with mCRPC who received standard-of-care 177Lu-PSMA-617 outside clinical trials. We assessed whether observed PSA responses and documented adverse events were consistent with trial findings. Treatment exposure and study-defined PFS were additional descriptive outcomes.

## 2. Materials and Methods

### 2.1. Study Design and Population

This retrospective study included all consecutive patients with PSMA-positive mCRPC who received at least one administration of standard-of-care 177Lu-PSMA-617 outside a clinical trial at Severance Hospital in Sinchon, Seoul, Republic of Korea. Thirteen patients were identified from institutional treatment records covering 2022 through 30 June 2026. Eligibility did not depend on treatment response or completion of six cycles. Eighteen patients who received LuPSMA within clinical trials were excluded because the study concerned treatment delivered as standard care.

PSMA imaging eligibility for treatment followed the VISION criteria [3]. Clinical and laboratory information, treatment administration dates, PSA measurements, imaging reports, and documented follow-up outcomes were reviewed retrospectively. The data cutoff was 30 June 2026.

### 2.2. Treatment

177Lu-PSMA-617 (Novartis, Basel, Switzerland) was administered intravenously at 200 mCi (7.4 GBq) per cycle according to the institutional protocol. Cycles were given at intervals of 6–9 weeks according to treatment scheduling and clinical status. Delivered cycles and activity reductions were recorded. Two patients received a reduced activity of 160 mCi (5.92 GBq) because of cytopenias.

Previous androgen-deprivation therapy (ADT), ARPI, and chemotherapy exposure were distinguished from treatment given concurrently with 177Lu-PSMA-617. Serum testosterone measurements during treatment were reviewed to establish castrate status. Concurrent non-ADT systemic therapy comprised systemic anticancer agents other than ADT administered between the first and last 177Lu-PSMA-617 administrations.

### 2.3. Assessments and Outcome Definitions

Age at treatment initiation was calculated in completed years from date of birth and the exact first administration date. The highest documented Gleason score was used. Time from documented mCRPC onset to treatment initiation was calculated at month-level precision. Baseline hemoglobin, platelet count, creatinine, and alkaline phosphatase were obtained from assessments before the first administration. Eastern Cooperative Oncology Group (ECOG) performance status could not be reliably ascertained and was not included.

Baseline PSA was the closest measurement on or before the first administration. PSA50 and PSA90 were defined as decreases of at least 50% and 90%, respectively, from this value at any available post-baseline assessment; confirmatory measurements were not required. Best percentage change was calculated as 100 × (lowest post-baseline PSA − baseline PSA)/baseline PSA. Available measurements during treatment and follow-up through the data cutoff were included; initiation of subsequent systemic therapy was not used to censor the PSA series. The cycle-linked assessments at which the PSA50 and PSA90 thresholds were first reached were also reviewed. Response proportions used all 13 patients as the denominator. PSA was assessed during routine care without a uniform sampling schedule. The longitudinal plot displays sequential assessments rather than equal calendar-time intervals.

Study-defined PFS was measured from the exact first administration date to the earliest documented radiographic progression, qualifying PSA progression, or death. Radiographic progression on whole-body bone scintigraphy, computed tomography (CT), or PSMA PET was counted according to the clinical report, without a hierarchy among modalities. PSA progression required a documented increase of at least 50% from the pretreatment baseline that was considered disease progression.

The first documented progression date was used rather than an imputed date between treatment cycles or imaging visits. If death preceded documented progression, death was the event. Patients without an event were censored on the last documented non-progression date; the last outpatient date was used for patients lost to follow-up. Follow-up was administratively censored at the data cutoff. Calendar-day intervals were converted to months using 365.25/12 days per month. Imaging was performed as part of routine care without a fixed schedule. The endpoint included PSA progression in addition to the radiographic progression or death used in VISION [3]; PSA, radiographic, and clinical outcomes are distinguished in Prostate Cancer Clinical Trials Working Group (PCWG) recommendations [2,19].

Adverse events documented during treatment were summarized by event type, patient count, and the highest recorded grade. Graded events were included when a Common Terminology Criteria for Adverse Events (CTCAE) grade was explicitly documented. Causality was not assigned retrospectively, and laboratory abnormalities were not converted to CTCAE grades post hoc. Cytopenias associated with activity reduction were reported separately. PSA increases were treated as efficacy observations rather than adverse events. Because ascertainment relied on routine records, absence of a documented event was not interpreted as evidence that it had not occurred.

Two anonymized patients who completed six cycles were selected after treatment to illustrate contrasting longitudinal courses. Their PSA measurements, imaging reports, recorded adverse events, and laboratory nadirs were described. These cases were not sampled to represent the cohort or to test whether toxicity predicted response.

### 2.4. Statistical Analysis

Continuous variables were summarized as medians and interquartile ranges (IQRs), and categorical variables as counts and percentages. Two-sided 95% Wilson score confidence intervals (CIs) were calculated for PSA50 and PSA90 proportions. The number of distinct documented adverse-event types per patient was summarized by PSA50 status without hypothesis testing.

PFS was estimated by the Kaplan–Meier method. Pointwise 95% CIs used Greenwood variance and a log-log transformation. The CI for median PFS was obtained by inversion of the survival-function CI; a bound that did not cross a survival probability of 0.5 was reported as not estimable. Given the cohort size and number of events, no multivariable adjustment, regimen comparison, or PFS subgroup hypothesis test was performed. Overall survival was not analyzed. Calculations were performed with Python version 3.14.4, lifelines version 0.30.3, and pandas version 2.3.3.

### 2.5. Ethics

The study protocol was approved by the Institutional Review Board of Severance Hospital, Yonsei University Health System (IRB No. 2026-1884-001; approval date: 15 July 2026). The requirement for informed consent was waived by the Institutional Review Board because of the retrospective nature of the study.

## 3. Results

### 3.1. Patient Characteristics and Prior Treatment

The cohort comprised all 13 eligible patients treated with 177Lu-PSMA-617 outside clinical trials. Median age at cycle 1 was 69 years (IQR, 67–71; range, 55–84). The median highest documented Gleason score was 9 (IQR, 8–9), and 12 patients (92.3%) had a highest score of at least 8. The median interval from the documented onset of mCRPC to cycle 1 was 30 months (IQR, 27–38). Median cycle 1 PSA was 88.79 ng/mL (IQR, 32.43–264.68). Pretreatment median hemoglobin was 10.9 g/dL, platelet count 161 × 10^9^/L, creatinine 0.74 mg/dL, and alkaline phosphatase 115 U/L. All 13 patients had received prior ADT, an ARPI, and docetaxel (Table 1).

### 3.2. Treatment Exposure and Concurrent Therapy

Patients received a median of four cycles (IQR, 2–6), and four (30.8%) completed six cycles. The standard activity was 200 mCi (7.4 GBq) per cycle. Two patients required reduction to 160 mCi (5.92 GBq) because of cytopenias; one received a transfusion before reduced-activity treatment. All 13 patients had documented castrate serum testosterone levels during 177Lu-PSMA-617 treatment. Concurrent non-ADT systemic therapy was documented in three patients (23.1%): enzalutamide in two (15.4%) and pembrolizumab in one (7.7%) (Table 1).

### 3.3. PSA Response

**Figure 1:**
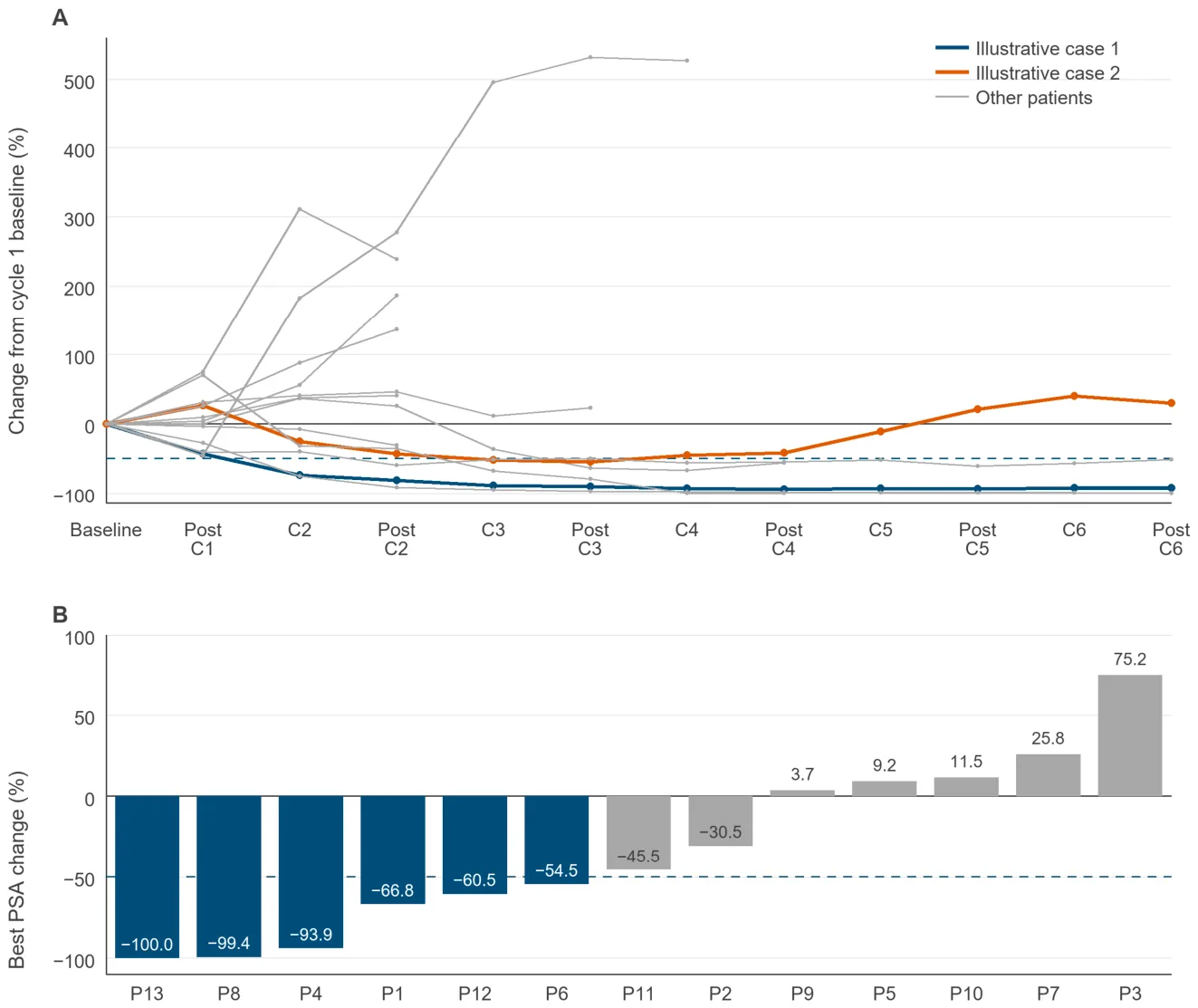
Prostate-specific antigen (PSA) kinetics during 177Lu-PSMA-617 treatment. (**A**) Longitudinal percentage change from the closest PSA measurement on or before cycle 1 for all 13 patients at sequential assessments during treatment and follow-up. Actual intervals varied. Blue and orange lines represent Cases 1 and 2, respectively; grey lines represent the other 11 patients. (**B**) Best post-baseline percentage change for each patient; blue bars indicate PSA50 responses and grey bars indicate changes that did not meet PSA50. Dashed blue lines indicate the PSA50 threshold (−50% from baseline); solid horizontal lines at 0% indicate no change. C, cycle; PSA50, PSA reduction of at least 50%; PSMA, prostate-specific membrane antigen.

PSA50 was observed in six patients (46.2%; Wilson 95% CI, 23.2–70.9), and PSA90 was observed in three (23.1%; Wilson 95% CI, 8.2–50.3). All six PSA50 responders, including the three PSA90 responders, first met the respective thresholds by their final treatment cycle. Figure 1A shows longitudinal percentage changes from the cycle 1 PSA for all 13 patients; the two illustrative cases are highlighted. Figure 1B shows the best post-baseline percentage change for each patient.

### 3.4. Progression-Free Survival

**Figure 2:**
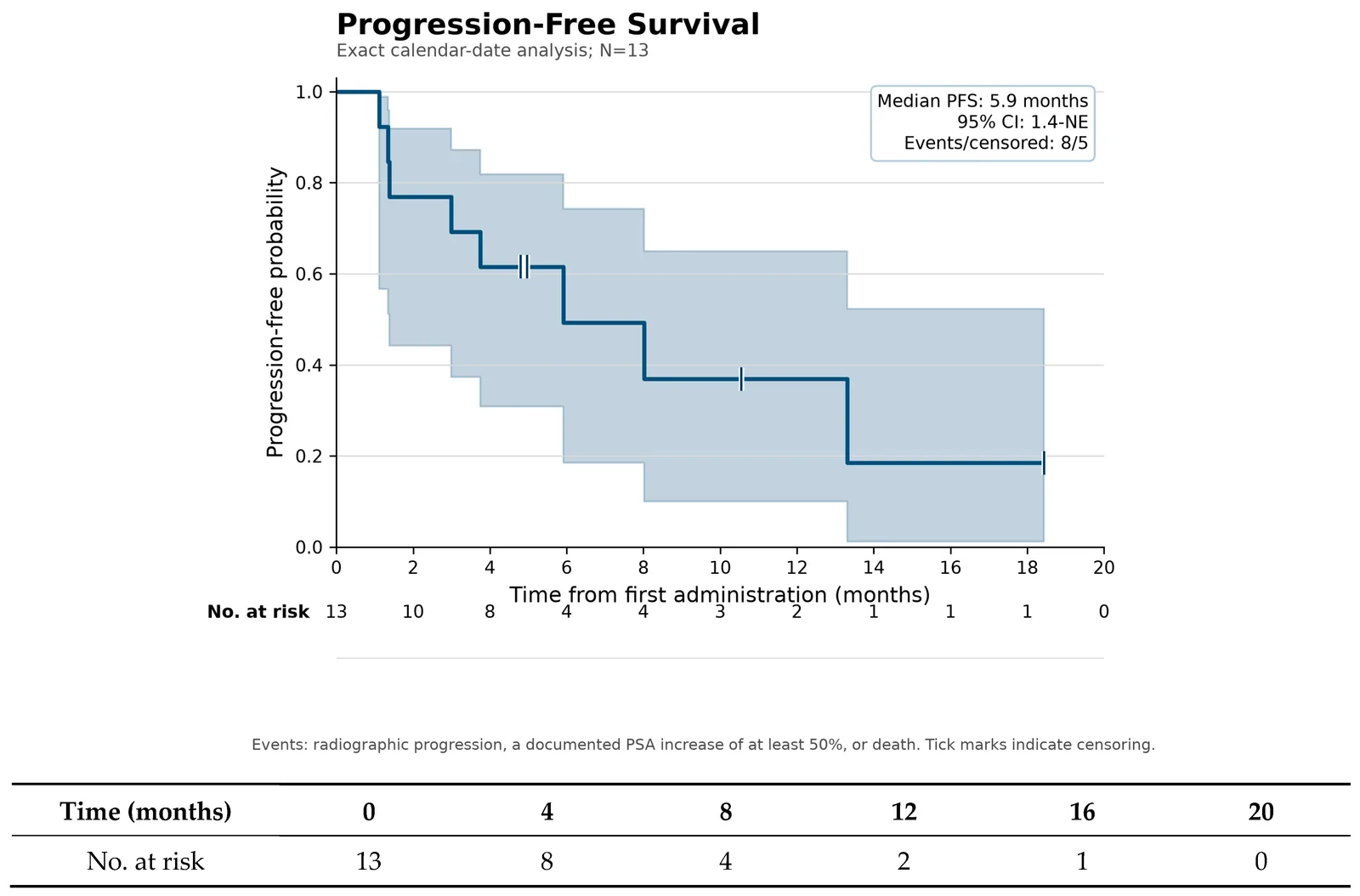
Kaplan–Meier estimate of study-defined progression-free survival (PFS) after 177Lu-PSMA-617. Time was calculated from the exact first administration date to radiographic progression, a documented PSA increase of at least 50% from pretreatment baseline that was considered disease progression, or death. Patients without an event were censored on the last documented non-progression date or, for loss to follow-up, the last outpatient date. Median PFS was 5.9 months (95% confidence interval (CI), 1.4 months to not estimable). The solid blue line represents the Kaplan–Meier estimate, and the shaded area indicates its 95% confidence interval. Tick marks indicate censoring. PSA, prostate-specific antigen; NE, not estimable.

Eight patients met the PFS event definition and five were censored. Median study-defined PFS was 5.9 months (95% CI, 1.4 months to not estimable). The numbers at risk at 0, 4, 8, 12, 16, and 20 months were 13, 8, 4, 2, 1, and 0, respectively (Figure 2).

### 3.5. Adverse Events

Adverse events documented during treatment are summarized in Table 2. Dry mouth was recorded in five patients (38.5%; maximum grade 2), back pain in two (15.4%; maximum grade 2), and paresthesia or limb tingling in two (15.4%), including one grade 3 event. Nausea, generalized weakness, anorexia, pelvic/groin pain, flank pain, lumbar pain, thrombocytopenia, and sepsis were each recorded in one patient (7.7%). The sepsis event was grade 3.

Causality to 177Lu-PSMA-617 was not adjudicated. Pain, neurologic symptoms, and sepsis could reflect the underlying malignancy or intercurrent illness. Laboratory abnormalities were not assigned CTCAE grades post hoc because measurement timing and baseline-to-nadir grading could not be reconstructed uniformly. Cytopenias requiring activity reduction are reported separately under treatment exposure. Ten patients (76.9%) had at least one graded adverse event documented in the medical record.

### 3.6. Illustrative Case Comparison

Among the four patients who completed all six cycles, two anonymized cases with contrasting longitudinal treatment courses were evaluated descriptively. Both patients received 200 mCi (7.4 GBq) per cycle at intervals of 6–9 weeks.

Case 1 was a 69-year-old man who had undergone radical prostatectomy for Gleason score 9 (5 + 4), pT3bN0 acinar adenocarcinoma and subsequently received salvage radiotherapy, ADT, 11 cycles of docetaxel, and abiraterone plus prednisone. He received six 200-mCi cycles at 6- to 9-week intervals. PSA declined from 88.79 ng/mL before cycle 1 to a nadir of 5.44 ng/mL (−93.9%) and remained substantially below baseline at 7.98 ng/mL on 15 June 2026. Serial PSMA PET reports described decreasing skeletal uptake consistent with a favorable treatment response. Grade 1 pelvic/groin pain was documented, although a treatment-related cause was not established. The lowest recorded hemoglobin and platelet values were 9.9 g/dL and 72 × 10^9^/L, respectively.

Case 2 was an 84-year-old man with Gleason score 8 (4 + 4), T4N0M1b acinar adenocarcinoma and multiple bone metastases at diagnosis. Previous treatment included ADT, abiraterone, chemotherapy, enzalutamide, and radiotherapy. PSA declined from 32.43 ng/mL at cycle 1 to 14.76 ng/mL (−54.5%) but subsequently increased to 45.40 ng/mL before cycle 6. Whole-body bone scintigraphy and PSMA PET documented osseous progression despite stable abdominal CT findings. Grade 2 dry mouth, grade 2 anorexia, grade 1 thrombocytopenia, and grade 1 distal paresthesia were recorded without adjudicated causality. The lowest recorded hemoglobin and platelet values were 12.3 g/dL and 53 × 10^9^/L. Olaparib was started after cycle 6 and before the 2 October 2025 assessment.

**Figure 3:**
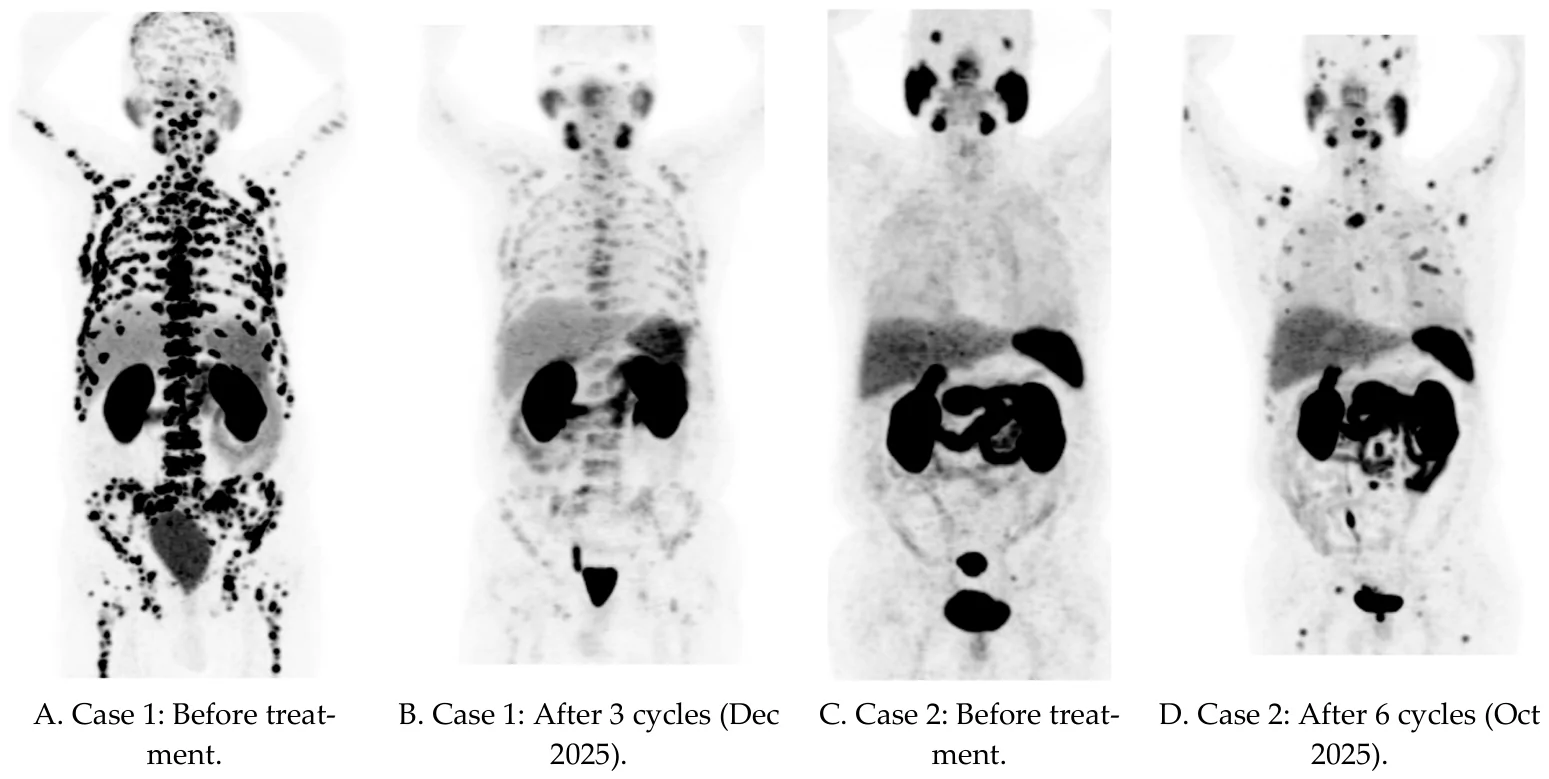
Prostate-specific membrane antigen (PSMA) positron emission tomography (PET) maximum-intensity-projection images in the two illustrative cases. (**A**) Case 1 before treatment. (**B**) Case 1 after three cycles, showing a qualitative decrease in PSMA-avid skeletal disease. (**C**) Case 2 before treatment. (**D**) Case 2 after six cycles on 2 October 2025, showing increased PSMA-avid skeletal disease. Olaparib had been started after cycle 6 and before panel (**D**). The panels are descriptive; acquisition timing and display scales differ, and the images were not used alone to assign a formal response category.

Case 1 had a sustained PSA decline and favorable serial imaging with one documented adverse-event type, whereas Case 2 had an initial PSA50 response followed by a PSA rebound and osseous progression with four documented event types. This comparison is descriptive. Across the full cohort, the median number of documented event types was one in both PSA50 responders and nonresponders; at least two event types were recorded in one of six responders and two of seven nonresponders. These descriptive observations cannot establish an association between adverse-event burden and PSA response (Table 3 and Table 4; Figure 3).

## 4. Discussion

PSA50 was observed in six of thirteen patients (46.2%), including three who achieved PSA90. These responses show that the biochemical activity reported in clinical trials was also observed in patients treated in routine practice at our institution. Treatment exposure and assessment schedules varied among patients. The study therefore contributes to post-approval validation by documenting PSA responses under the conditions in which 177Lu-PSMA-617 is used outside trials.

The PSA50 proportion was within the range reported in larger post-approval cohorts. Gafita et al. reported PSA50 in 30 of 74 evaluable patients [16], and Moradi Tuchayi et al. reported PSA50 in 60 of 99 patients [17]. Multicenter studies and prospective registries have also documented biochemical activity [14,15]. The wide CI for our estimate (23.2–70.9%) and differences in selection, treatment exposure, and assessment schedules limit comparisons across cohorts. A larger real-world comparison with cabazitaxel was reported in the ARON-3 study [20].

PSA decline was associated with clinical outcomes in VISION [21], and studies of abiraterone and radium-223 have examined the prognostic relevance of early PSA changes [22,23]. In this series, response was based on the lowest available PSA during treatment and follow-up, without required confirmation. All PSA50 and PSA90 thresholds were first reached by the final treatment cycle. Thus, the response classifications did not depend on measurements obtained only after completion of 177Lu-PSMA-617 treatment. Concomitant systemic treatment may nevertheless have contributed to PSA changes.

Median study-defined PFS was 5.9 months. The endpoint included a PSA increase of at least 50% from pretreatment baseline, radiographic progression on bone scintigraphy, CT, or PSMA PET, or death. A baseline-referenced PSA threshold may not capture a rise from nadir that remains below baseline, while a qualifying rise may precede imaging evidence of progression. These differences, together with nonuniform imaging, restrict direct comparison with VISION imaging-based PFS. PCWG recommendations distinguish disease manifestations and assessment rules for this reason [2,19]. The first documented progression date still depended on the timing of assessments. The non-estimable upper CI bound reflects limited information from eight events and censored follow-up.

Dry mouth was the most frequently documented adverse event, and cytopenias required activity reduction in two patients. Both have been reported with 177Lu-PSMA-617 in VISION [3]. These observations are consistent with recognized toxicities, although routine records may not capture adverse events as completely as trial-based assessments. Previous studies and the VISION dosimetry substudy have examined hematologic and renal safety [24,25,26]. Baseline anemia and previous chemotherapy make hematologic monitoring relevant in this setting. Procedure guidance recommends serial blood counts and renal-function assessment [27].

All patients had received ADT, an ARPI, and docetaxel, but their prior treatment sequences and duration differed. Castrate testosterone levels were documented in all patients during 177Lu-PSMA-617 treatment. Concurrent non-ADT therapy comprised enzalutamide in two patients and pembrolizumab in one. A retrospective series of 108 patients did not find an independent association between concurrent ARPI use and improved outcomes [28]. The contribution of concurrent treatment in our cohort remains uncertain. Delivered cycles also depend on response, progression, and tolerability, as well as the ability to continue treatment. Cycle count was therefore reported descriptively.

The illustrative cases show different courses after an initial PSA50 response. Case 1 had a sustained PSA decline with favorable serial imaging, whereas Case 2 developed a PSA rebound and osseous progression. The latter had more documented clinical adverse-event types, but the median number of documented event types was one in both PSA50 responders and nonresponders. These selected cases illustrate clinical variability; they cannot establish whether adverse-event burden predicts response. The later PET assessment in Case 2 also followed initiation of olaparib.

Clinical prognostic models and VISION analyses have evaluated baseline disease characteristics and quantitative PSMA PET findings [29,30,31]. Imaging eligibility studies and early post-treatment single-photon emission computed tomography (SPECT)/CT assessments have further examined selection and response evaluation [32,33,34]. Incorporating such measures into prospective routine-care data collection could help explain differences in response durability.

This study is limited by its small sample, retrospective single-center design, and absence of a comparator. ECOG performance status was unavailable, and previous and concurrent therapies were heterogeneous. The number of cycles and available PSA measurements differed among patients, affecting opportunities to observe a response. Later PSA measurements were not censored at the start of subsequent systemic therapy. Imaging was not scheduled uniformly, and the PSA-inclusive PFS endpoint differs from trial radiographic PFS. Adverse-event capture depended on routine documentation; causality was not adjudicated, and laboratory toxicity grades could not be reconstructed uniformly. These factors limit generalizability and attribution of efficacy and safety outcomes to 177Lu-PSMA-617 alone.

Future multicenter studies should assess response durability using prespecified biochemical and radiographic endpoints. Standardized assessment intervals, serial laboratory measurements, and documentation of concurrent therapy and treatment discontinuation would also improve interpretation of outcomes and adverse events in routine practice.

## 5. Conclusions

PSA50 was observed in six of thirteen heavily pretreated patients, including three who achieved PSA90. The findings support the applicability of trial-reported PSA activity to routine 177Lu-PSMA-617 treatment. Documented adverse events included dry mouth and cytopenias requiring activity reduction, consistent with recognized toxicities. Response and adverse-event frequencies should be interpreted in light of the small cohort and retrospective ascertainment. Study-defined PFS included PSA-based progression and therefore differs from trial radiographic PFS. Larger prospective cohorts with standardized follow-up are needed to estimate response durability and adverse-event incidence more precisely.

## Figures and Tables

**Table 1:** Baseline and treatment characteristics of the 13-patient cohort.

Characteristic	Value
Age at cycle 1, median (IQR), years	69 (67–71)
Highest documented Gleason score, median (IQR)	9 (8–9)
Highest Gleason score ≥ 8, *n* (%)	12 (92.3)
Interval from documented onset of mCRPC to cycle 1, median (IQR), months	30 (27–38)
PSA at cycle 1, median (IQR), ng/mL	88.79 (32.43–264.68)
Baseline hemoglobin, median (IQR), g/dL	10.9 (10.3–12.7)
Pretreatment platelet count, median (IQR), ×10^9^/L	161 (145–248)
Baseline serum creatinine, median (IQR), mg/dL	0.74 (0.61–0.90)
Baseline alkaline phosphatase, median (IQR), U/L	115 (77–164)
177Lu-PSMA-617 cycles, median (IQR)	4 (2–6)
Completed six cycles, *n* (%)	4 (30.8)
Prior ADT, *n* (%)	13 (100.0)
Prior docetaxel, *n* (%)	13 (100.0)
Prior radiotherapy, *n* (%)	12 (92.3)
Prior radical prostatectomy, *n* (%)	8 (61.5)
Prior ARPI, *n* (%)	13 (100.0)
Documented castrate serum testosterone level, *n* (%)	13 (100.0)
Any concurrent non-ADT systemic therapy, *n* (%)	3 (23.1)
Concurrent enzalutamide, *n* (%)	2 (15.4)
Concurrent pembrolizumab, *n* (%)	1 (7.7)

Abbreviations: ADT, androgen-deprivation therapy; ARPI, androgen receptor pathway inhibitor; IQR, interquartile range; mCRPC, metastatic castration-resistant prostate cancer; PSA, prostate-specific antigen; PSMA, prostate-specific membrane antigen. Age was calculated in completed years from date of birth to the exact first 177Lu-PSMA-617 administration date. The mCRPC interval was calculated from the documented onset of castration-resistant progression; month-level dates were used when exact dates were unavailable. Baseline laboratory values were obtained from assessments performed before the first administration. All patients had documented castrate serum testosterone levels during treatment. Concurrent non-ADT systemic therapy comprised enzalutamide (*n* = 2) and pembrolizumab (*n* = 1).

**Table 2:** Adverse events documented during 177Lu-PSMA-617 treatment among 13 patients.

Adverse Event	Any Grade, *n* (%)	Grade 3–4, *n* (%)	Maximum Grade
Dry mouth	5 (38.5)	0	2
Back pain	2 (15.4)	0	2
Paresthesia or limb tingling	2 (15.4)	1 (7.7)	3
Pelvic/groin pain	1 (7.7)	0	1
Flank pain	1 (7.7)	0	1
Lumbar pain	1 (7.7)	0	1
Nausea	1 (7.7)	0	2
Generalized weakness	1 (7.7)	0	2
Anorexia	1 (7.7)	0	2
Thrombocytopenia	1 (7.7)	0	1
Sepsis	1 (7.7)	1 (7.7)	3

Events were included when a Common Terminology Criteria for Adverse Events (CTCAE) grade was explicitly recorded during treatment. Causality to 177Lu-PSMA-617 was not adjudicated. Prostate-specific antigen (PSA) increases were treated as efficacy observations and were not included as adverse events. Laboratory-only abnormalities were not assigned CTCAE grades post hoc.

**Table 3:** Longitudinal PSA values, measurement dates, and percent changes in the two illustrative cases.

Treatment Point	Case 1 PSA, ng/mL (Date)	Change from Baseline	Case 2 PSA, ng/mL (Date)	Change from Baseline
Cycle 1 baseline	88.79 (25 July 2025)	Reference	32.43 (2 January 2025)	Reference
Second cycle	23.11 (23 September 2025)	−74.0%	24.17 (13 February 2025)	−25.5%
Third cycle	10.23 (17 November 2025)	−88.5%	15.48 (27 March 2025)	−52.3%
Fourth cycle	6.10 (12 January 2026)	−93.1%	17.85 (8 May 2025)	−45.0%
Nadir	5.44 (19 February 2026)	−93.9%	14.76 (18 April 2025)	−54.5%
Fifth cycle	6.17 (17 March 2026)	−93.1%	28.84 (3 July 2025)	−11.1%
Sixth cycle	7.13 (12 May 2026)	−92.0%	45.40 (3 September 2025)	+40.0%
Last documented follow-up after cycle 6	7.98 (15 June 2026)	−91.0%	42.15 (2 October 2025)	+30.0%

Percent change was calculated from the closest prostate-specific antigen (PSA) value obtained on or before the first administration. In Case 1, baseline PSA was measured 4 days before cycle 1; in Case 2, it was measured on the cycle 1 date. The post-cycle-6 row reports the last verified PSA value available during follow-up.

**Table 4:** Adverse events and laboratory nadirs in the two illustrative cases.

Finding	Case 1	Case 2
Pelvic/groin pain	Grade 1	Not recorded
Dry mouth	Not recorded	Grade 2
Anorexia	Not recorded	Grade 2
Thrombocytopenia	Not reported	Grade 1
Distal paresthesia	Not recorded	Grade 1
Lowest recorded hemoglobin	9.9 g/dL	12.3 g/dL
Lowest recorded platelet count	72 × 10^9^/L	53 × 10^9^/L

Adverse-event grades are presented without attribution of causality. Laboratory nadirs are descriptive and were not graded post hoc.

## Data Availability

Patient-level data are not publicly available because they contain protected clinical information. Access to de-identified data is subject to institutional and IRB approval.
